# Toward Renewable Solar Energy Systems: Advances in Photocatalytic Green Hydrogen Production

**DOI:** 10.1002/gch2.202400122

**Published:** 2024-05-27

**Authors:** Pablo Jiménez‐Calvo, Oleksandr Savateev, Katherine Villa, Mario J. Muñoz‐Batista, Kazunari Domen

**Affiliations:** ^1^ Chemistry of Thin Film Materials IZNF Friedrich‐Alexander‐Universität Erlangen‐Nürnberg Cauerstraße 3 91058 Erlangen Germany; ^2^ Department of Chemistry The Chinese University of Hong Kong Shatin, New Territories Hong Kong P. R. China; ^3^ Institute of Chemical Research of Catalonia (ICIQ) The Barcelona Institute of Science and Technology (BIST) Av. Països Catalans, 16 Tarragona 43007 Spain; ^4^ Department of Chemical Engineering University of Granada Granada 18074 Spain; ^5^ Research Initiative for Supra‐Materials Interdisciplinary Cluster for Cutting Edge Research Shinshu University Nagano 380‐8553 Japan; ^6^ Office of University Professors The University of Tokyo Bunkyo‐ku Tokyo 113‐8656 Japan

Green hydrogen (H_2_) production is relevant to sustainable energy systems due to its potential to decarbonize various sectors and mitigate climate change. Our inspiration draws from nature.

In fact, plant life has been inspiring human innovation for centuries. Plants’ ability to convert solar energy into chemical energy, as well as their autonomous smart functioning, are key reasons for this inspiration. Natural photosynthesis remains the core focus in our quest to understand its mechanisms and apply its principles to artificial systems.

Artificial photosynthesis plays a crucial role in addressing global challenges related to energy sustainability and environmental conservation. By mimicking natural photosynthesis, it offers a promising avenue for renewable energy generation, notably through H_2_ fuel production from water splitting. This technology provides clean energy and turns carbon dioxide into useful fuels and chemicals, cutting greenhouse gas emissions and fighting climate change. Moreover, it can transform agriculture by enabling simpler production of fertilizers and other compounds of interest. Thus, the development of more efficient artificial photosynthetic systems has the potential to help achieving carbon‐neutrality.

This special issue (SI) entitled “*Toward Renewable Solar Energy Systems: Advances in Photocatalytic Green Hydrogen Production*” was guest edited by Pablo Jiménez Calvo, Oleksandr Savateev, Katherine Villa, Mario J. Muñoz‐Batista, and Kazunari Domen. The aim and scope of this SI are to offer an updated overview of recent advances in various aspects of H_2_ technology: materials, devices, and technological innovations, thereby advancing toward the goal of a circular economy based on sustainable energy systems. The contributions comprise a diverse range of research, reviews, and perspective articles, centered in the green H_2_ production theme.^[^ ^1^ ^]^

This SI aims to address several key objectives in the field of H_2_ production employing different technologies driven by artificial solar‐light as an energy source. First, it focuses on the development and design of innovative materials and systems geared toward enhancing the efficiency of H_2_ generation through mainly photocatalysis, in lesser extent through photoelectrocatalysis and photoreforming. Second, some studies delve into the complexities surrounding the scaling up of photocatalytic H_2_ production, examining both the challenges and opportunities in transitioning from laboratory to pilot devices. Third, the reviews scrutinize the value chain and direct photocatalytic conversion of green H_2_ into high added‐value chemicals, as a solar to chemical strategy to diversify the utilization of H_2_.

The contributions offer diverse viewpoints from researchers across Latin America, Europe, and Asia, who are established experts and up‐and‐coming researchers in the field of solar energy research. The discussions center around photoelectrocatalysis, photocatalysis, and electrocatalysis as solar‐driven water splitting processes. Importantly, PV‐driven water splitting has achieved a relatively high Technological Readiness Level (TRL 7), reaching solar‐to‐H_2_ efficiencies of up to 20%.^[^ ^2^ ^]^ Yet, mentioned technologies in this context have also shown promising efficiencies, each with its own set of advantages and limitations.

Ever since the pioneering work by Fujishima and Honda in 1972 on photocatalytic H_2_O splitting using TiO_2_,^[^ ^3^ ^]^ vast efforts focused on exploring high‐efficiency light‐responsive (photo‐) or (electro‐)catalysts for a broad range of energy‐ and environmental‐applications. This includes water splitting to H_2_ and oxygen, reduction of CO_2_ to high‐energy >C1 products, nitrogen fixation to ammonia, degradation of pollutants, organic transformation, and others.

The subsequent paragraphs will provide a summary of the papers featured in this SI, serving as an overview for the wide and technical readership.

For example, Wong et al have investigated a cutting‐edge hybrid composite, comprising crystalline carbon nitride (CCN) and Ti_3_C_2_T_x_ MXene (https://doi.org/10.1002/gch2.202300235). Their method consists of a combination of salt‐assisted and freeze‐drying steps to ensure a close interface between the two components. The CN community actively seeks to enhance the photoactivity of graphitic CN by improving its crystallinity, including reducing structural defects. This is achieved by incorporating metal cations into the structure, stabilized by surrounding nitrogen bridge atoms, forming distinct structural pores.

The synthesis strategy varies the MXene loading counterpart ranging from 0.2 up to 10 wt.%. The resulting CCN/TCT‐0.5/Pt hybrid achieved remarkable 7.3% of apparent quantum efficiency at 420 nm and an elevated H_2_ evolution rate of 2652 µmol h^−1^ g^−1^, outperforming all its homologs. The enhanced activity was attributed to the combination of Ti_3_C_2_T_x_’s excellent conductivity, the abundant active sites provided by Ti_3_C_2_T_x_ and Pt, and the strong interfacial contact established between CCN and Ti_3_C_2_T_x_. Thus, this study serves as an example of how the formation of heterojunctions, coupling two individual semiconductors, leads to a significant improvement and, most importantly, enhances our understanding of photocatalytic systems. This insight can guide further research efforts in the design of more active catalysts.

Paineau et al introduced a rising star photoelectrocatalyst, 1D imogolite nanotubes (INTs), with relevant potential for H_2_ generation (https://doi.org/10.1002/gch2.202300255). While INTs have traditionally been used for dye and degradation reactions, a recent study demonstrated their remarkable ability to produce H_2_ at 1.5 mmol g^−1^ with 0.4 wt.% Ti incorporation in methanolic aqueous solution at full spectrum Xenon lamp irradiation. Similarly, a hybrid of 20% Au/INT‐CH_3_ and propan‐2‐ol (20%) as sacrificial agent, combined with monochromatic light of 254 nm assisted by photolysis and radiolysis yielded comparable H_2_ generation rates. These case studies serve as proof‐of‐concept for INTs as strong candidates for semiconductor catalysts, attributed to their well‐defined porous structure and permanent intra‐wall polarization resulting from bifunctional surfaces on the inner and outer tube walls.

Savateev et al discussed a method of producing H_2_ and acetal upon ethanol photoreforming (https://doi.org/10.1002/gch2.202300078). Converting bioethanol into 1,1‐diethoxyethane while simultaneously generating H_2_ enhances the economic viability of photocatalysis, diverging from the conventional approach of using nonselective oxidation of sacrificial agents for H_2_ production. The advantages of this reaction include the synthesis of 1 g of H_2_ along with 118 g of an organic molecule. This molecule can be used as an oxygenated additive for diesel, solvent, protecting group in organic synthesis, or fragrance.

Ionic carbon nitrides, such as potassium polyheptazine imide, are highlighted as promising semiconductors capable of cleaving the α‐H of ethanol to produce ketyl radicals. This is due to their potential in the valence band (+2.3 V versus NHE), conduction band (−0.1…−0.5 V versus NHE), basic surface (pKa ≈7), abundance of electron‐deficient heterocycles on sp^2^ N atoms, and electron‐deficient conjugated systems with temporary electron storage capacity. Economic analysis indicates a potential profit of 1000 euros when converting 8.7 L of ethanol into 7.2 L of acetal and 100 g of H_2_. This financial indicator validates this method as an alternative route for H_2_ and acetal simultaneous production and C_2_ utilization.

In their recent contribution, Sportelli et al. discussed the role of photoelectrocatalysis as an advance technology for H_2_ evolution (https://doi.org/10.1002/gch2.202400012). Addressing the common drawbacks of heterogeneous photocatalysis, namely fast charge recombination and low solar‐to‐H_2_ efficiency, photoelectrochemistry offer a solution by using an external circuit to separate the photogenerated charge carriers. Indeed, coupling H_2_ generation with direct in situ hydrogenation holds promise in addressing challenges related to H_2_ storage and distribution.

The justification of photoelectrocatalysis is rooted in addressing one of the key impediments facing the acceptance of the “hydrogen economy” – safety concerns. Traditional H_2_ storage methods involve pressurized tanks, posing risks due to H_2_’s high flammability and potential for explosion. Alongside liquid H_2_ carriers and solid‐state storage, in situ hydrogenation is a third viable avenue for obtaining fine chemicals.

In the following review contribution, Diab et al have pointed why H_2_ should not only be viewed as a fuel but also as a valuable building block for the production of various molecules with added value across multiple industries (https://doi.org/10.1002/gch2.202300185). This review summarized the multiple uses, mostly at room temperature, and versatile potential of photocatalytic processes as a replacement for fossil‐derived H_2_.

Photocatalysis is relevant in solar‐to‐chemical reactions, facilitating the production of key molecules such as ammonia, hydrogen peroxide, reduced organic compounds via hydrogenation, formic acid, methanol, ethylene, methane, ethanol, carbon monoxide, acetic acid, ethane, formaldehyde, and ethylene through CO_2_ reduction. Contrarily, the existing catalytic processes, natural gas/methane reforming, CO_2_ hydrogenation, Fischer‐Tropsch, water‐gas‐shift, and reverse‐water‐gas‐shift, to name a few, unfortunately depend on expensive supported metals catalysts operating under harsh conditions.^[^ ^4^ ^]^ This work demonstrates how photocatalysis can shape the future of energy supply, acting as a crucial producer of intermediate molecules with the potential to transform the chemical industry toward a low‐carbon and net‐zero emissions one.

In a similar vein, Garcia‐Navarro et al. conducted an analysis of the production, storage, distribution, and consumption of H_2_, offering a comprehensive roadmap to address existing bottlenecks (https://doi.org/10.1002/gch2.202300073). However, the competitiveness of H_2_ hinges on its price per unit mass, with widespread acceptance projected once it reaches $2/kg. In the energy landscape, H_2_ is envisioned as a central clean energy source for generating electricity, heat, and power. While the interconnectivity of the H_2_ value chain is well‐defined, storage and distribution lag behind production advances. Consequently, infrastructure development, mobility, battery technology, and decentralized stations remain key topics of discussion.

This review focuses on Green H_2_, which is generated using water, sunlight, and electricity from renewable sources. Three technologies meet this criterion: photocatalytic and photoelectrochemical water splitting, and photovoltaics combined with electrolysis at different efficiencies. Specifically, the common production methods of H_2_ in an electric circuit of an electrolytic cell are alkaline, proton exchange membrane, and solid‐oxide electrolysis cell. As of 2020, global electrolyzer manufacturing capacity was 20 MW year^−1^, with Europe aiming to reach 8 GW by 2030. Disparities between current capacities and future goals need to be carefully analyzed to inform collective actions. While photocatalysis shows promise alongside photoelectrochemical methods, solar‐to‐H_2_ efficiency still remains low at 1%.

In summary, photocatalysis holds the potential to revolutionize the energy sector but efficiencies need to be increased as well as the stability of the photocatalytic materials over long‐term reactions. As the transition from fossil to solar fuels gains momentum, key stakeholders can propose strategies and implement smart technological solutions. Thus, photocatalysis has the opportunity to advance its technological readiness level and establish itself as a feasible, efficient, clean, and sustainable energy solution in the near future.

Finally, green H_2_ production can promote energy independence and security by reducing reliance on imported fossil fuels. It also opens up new economic opportunities, creating jobs and stimulating innovation in the renewable energy sector. Overall, green H_2_ production is key for transitioning to a low‐carbon economy and achieving global climate goals.

## Conflict of Interest

The authors declare no conflict of interest.
